# Supplementary material for: (±)-Cryptamides A–D, Four Pairs of Novel Dopamine Enantiomer Trimers from the Periostracum Cicadae
Source: Molecules. 2022 Oct 9;27(19):6707. doi: 10.3390/molecules27196707 (PMC9571589; doi:10.3390/molecules27196707)
Supplement: Supplementary file 1 [file molecules-27-06707-s001.zip › molecules-1861142-supplementary.pdf]

molecules

Supplementary Materials

(±)-Cryptamides A–D, Four Pairs of Novel Dopamine Enantiomer Trimers from the Periostracum Cicadae

Junjian Luo [1,†], Wenjun Wei [1,†], Pan Wang [2], Tao Guo [1,*], Suiqing Chen [1,*], Liping Zhang[1] and Shuying Feng [3,*]

[1] School of Pharmacy, Henan University of Chinese Medicine, Zhengzhou 450046, China
[2] Academy of Chinese Medical Sciences, Henan University of Chinese Medicine, Zhengzhou 450046, China
[3] Medical College, Henan University of Chinese Medicine, Zhengzhou 450046, China
* Correspondence: gt010010@163.com (T.G.); suiqingchen@163.com (S.C.); fsy@hactcm.edu.cn (S.F.)
† These authors contributed equally to this work.

Computational Analysis

Table S1. Energy analysis for **1**.

Conformer	Gibbs free energy (298.15 K)		
	G (Hartree)	ΔE (kcal/mol)	Population (%)
1a	-2003.2431	0.0000	99.04
1b	-2003.2383	3.0160	0.61
1c	-2003.2378	3.3405	0.35

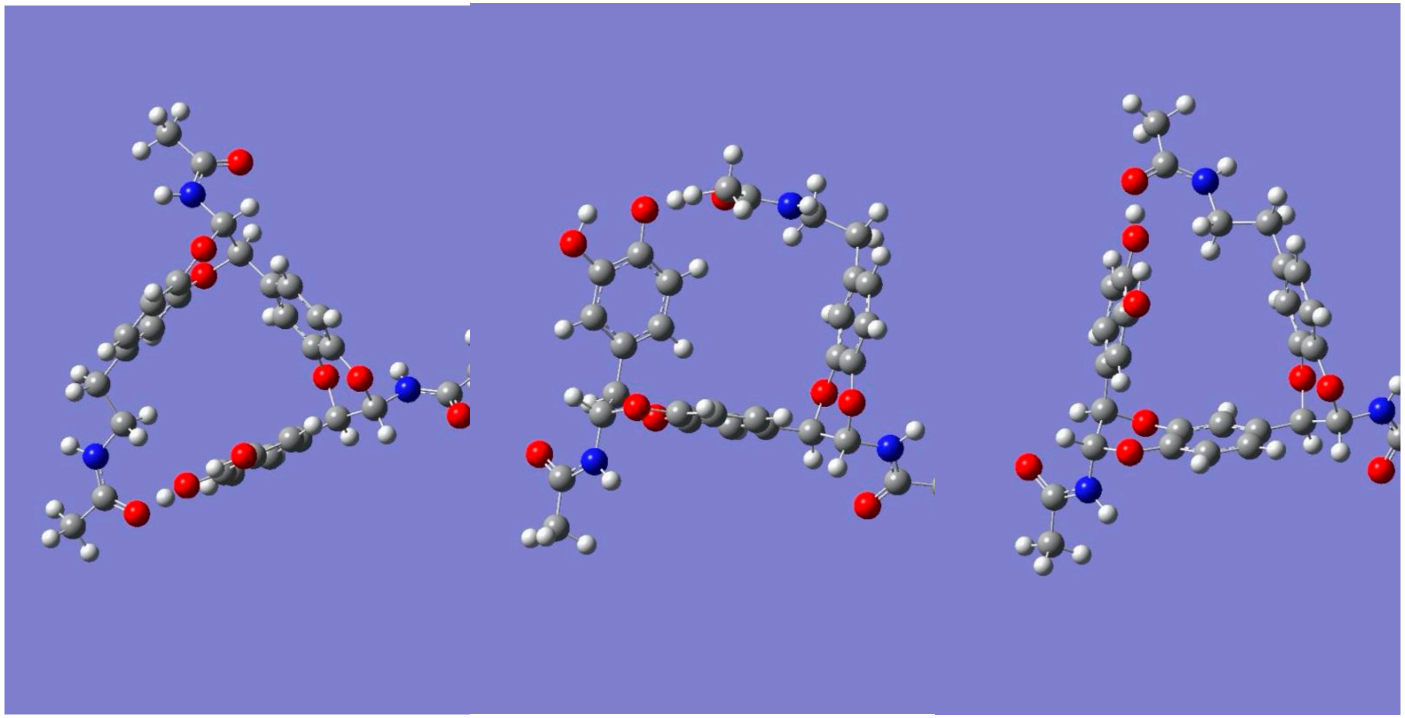

1a **1b** **1c**

Table S2. Energy analysis for **2**.

Conformer	Gibbs free energy (298.15 K)		
	G (Hartree)	ΔE (kcal/mol)	Population (%)
2a	-2003.2384	0.0000	100
2b	-2003.2278	6.6484	0

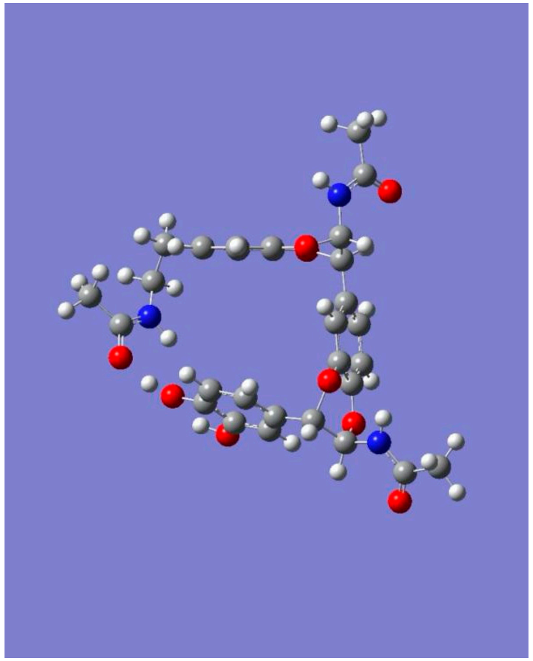

2a

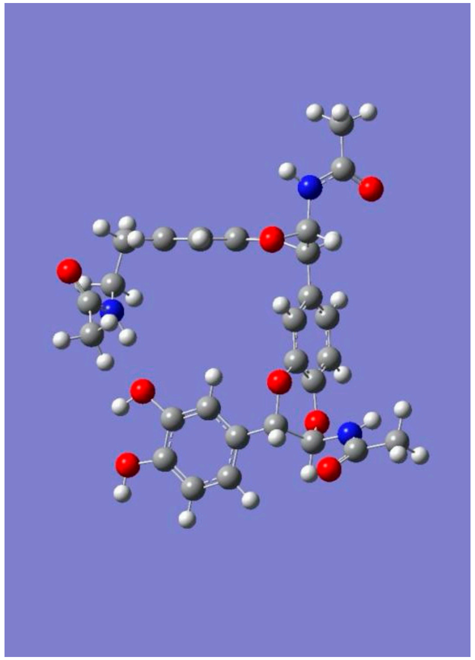

2b

Table S3. Energy analysis for **3**.

Conformer	Gibbs free energy (298.15 K)		
	G (Hartree)	ΔE (kcal/mol)	Population (%)
3a	-2003.2445	0.0000	99.92
3b	-2003.2364	5.0774	0.02
3c	-2003.2376	4.3398	0.07

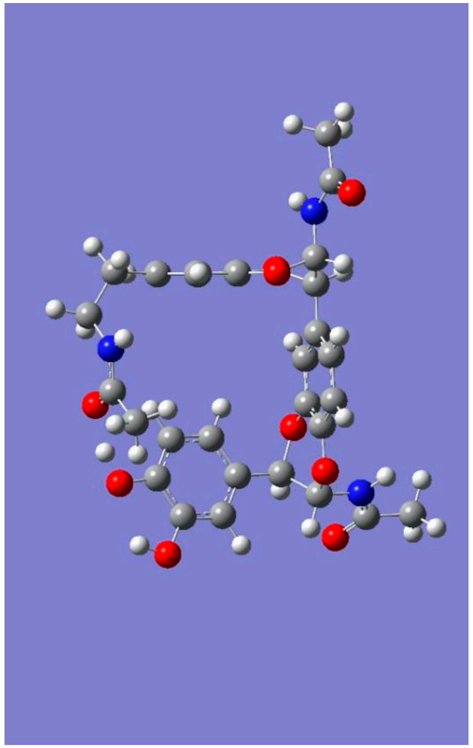

3a

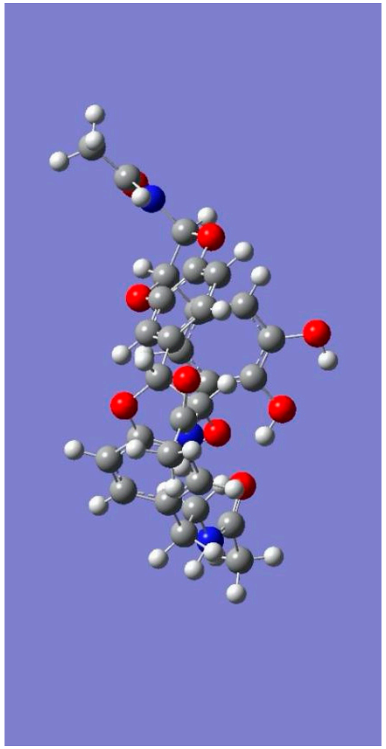

3b

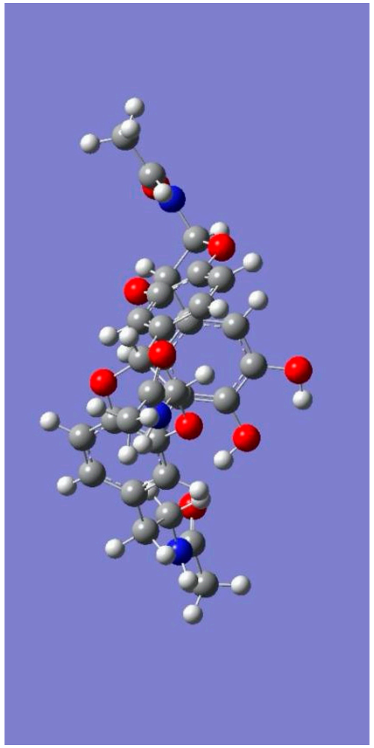

3c

Table S4. Energy analysis for **4**.

Conformer	Gibbs free energy (298.15 K)		
	G (Hartree)	ΔE (kcal/mol)	Population (%)
4a	-2003.2441	0.1745	42.68
4b	-2003.2444	0.0000	57.32

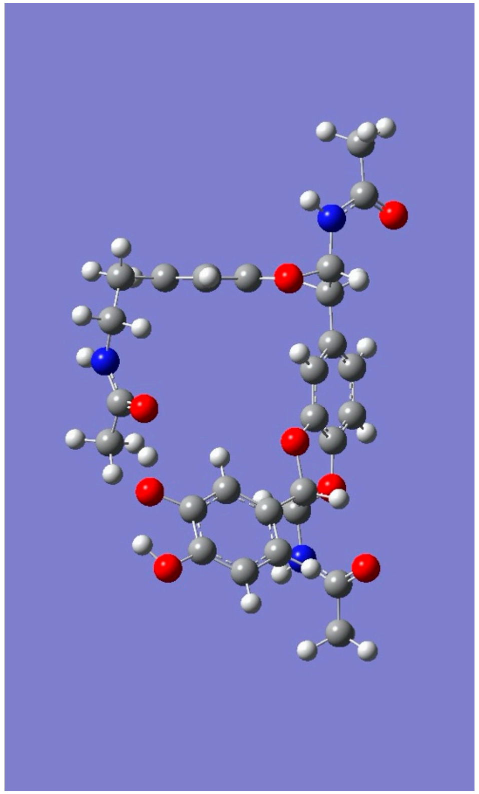

4a

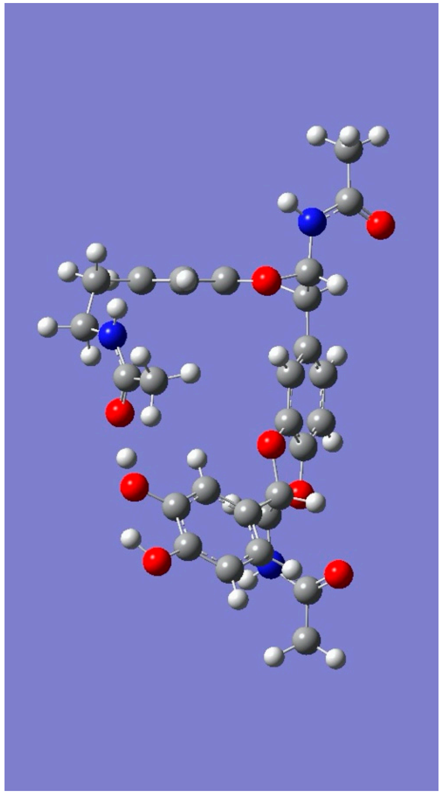

4b

NMR spectra of compounds **1–4**

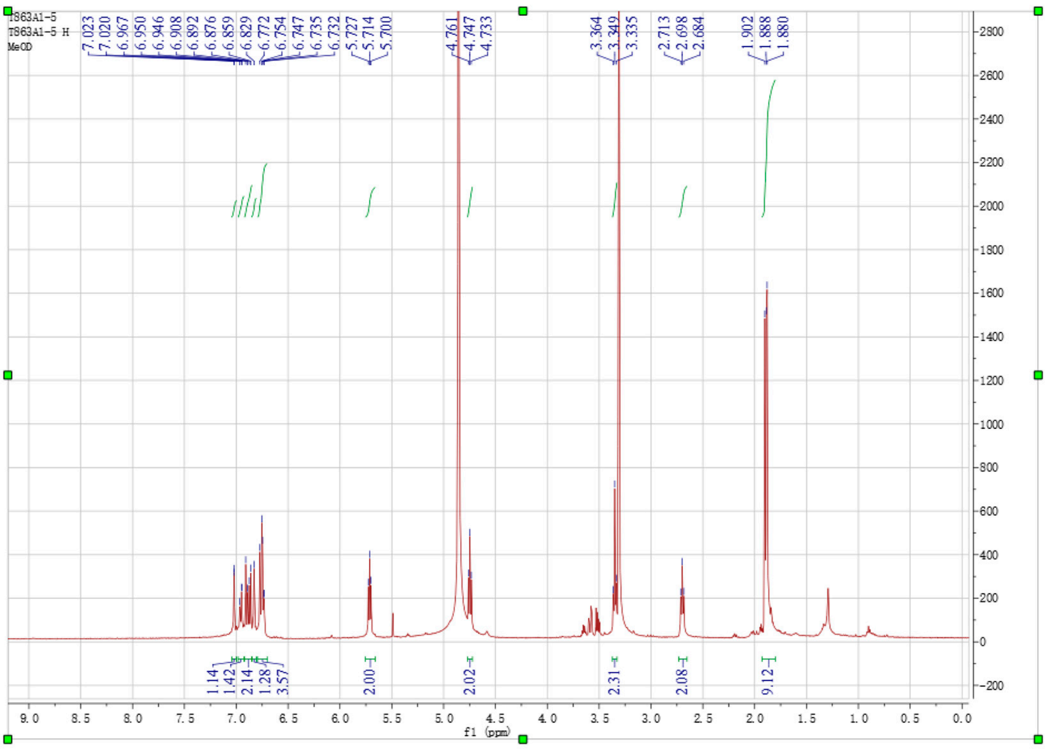

Figure S1. ^{1}H-NMR spectrum of **1** in methanol-d_4.

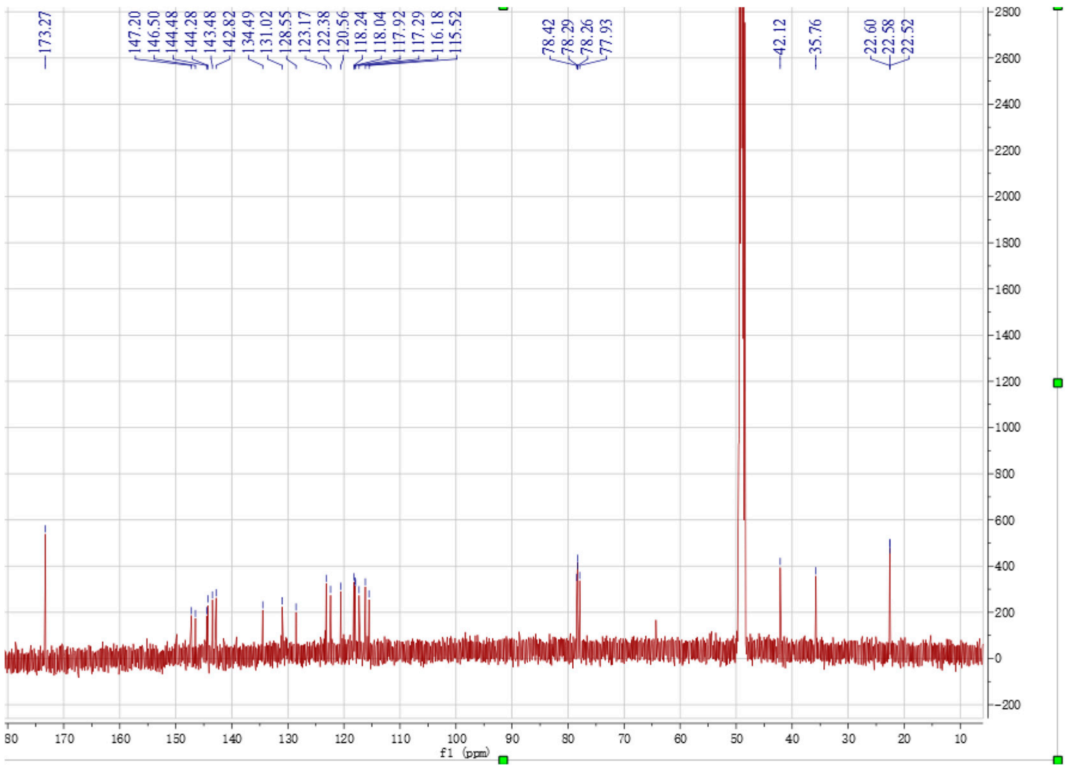

Figure S2. ^{13}C-NMR spectrum of **1** in methanol-d_4.

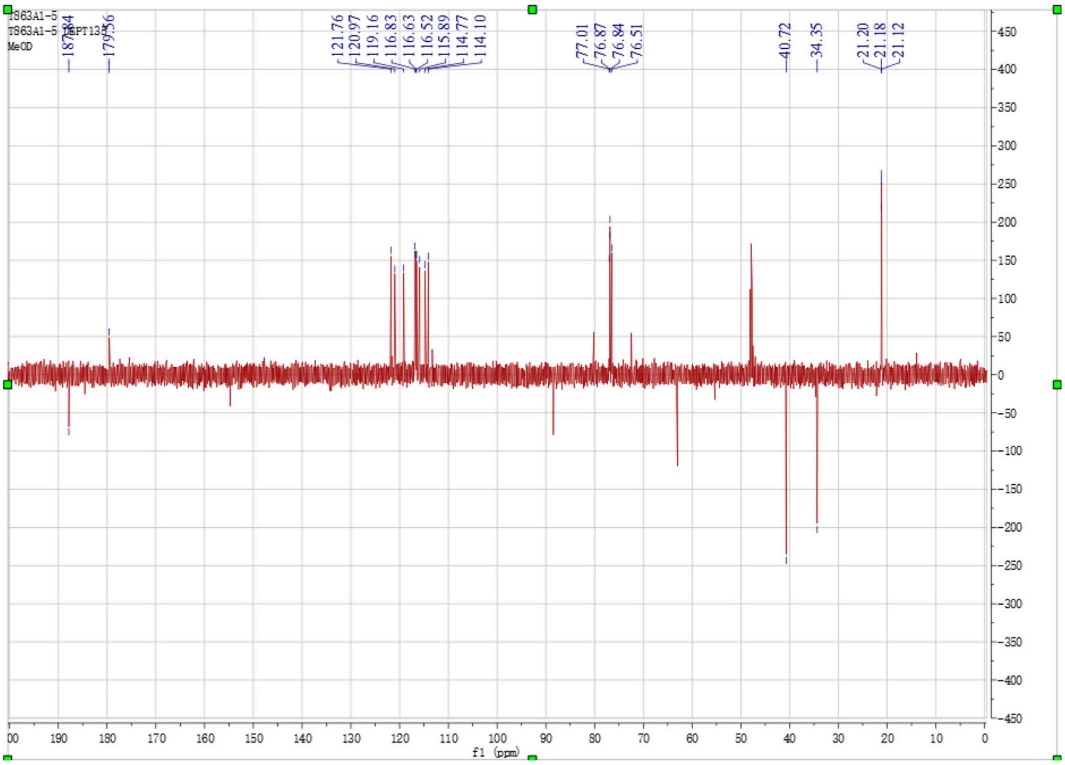

Figure S3. DEPT spectrum of **1** in methanol-d_4.

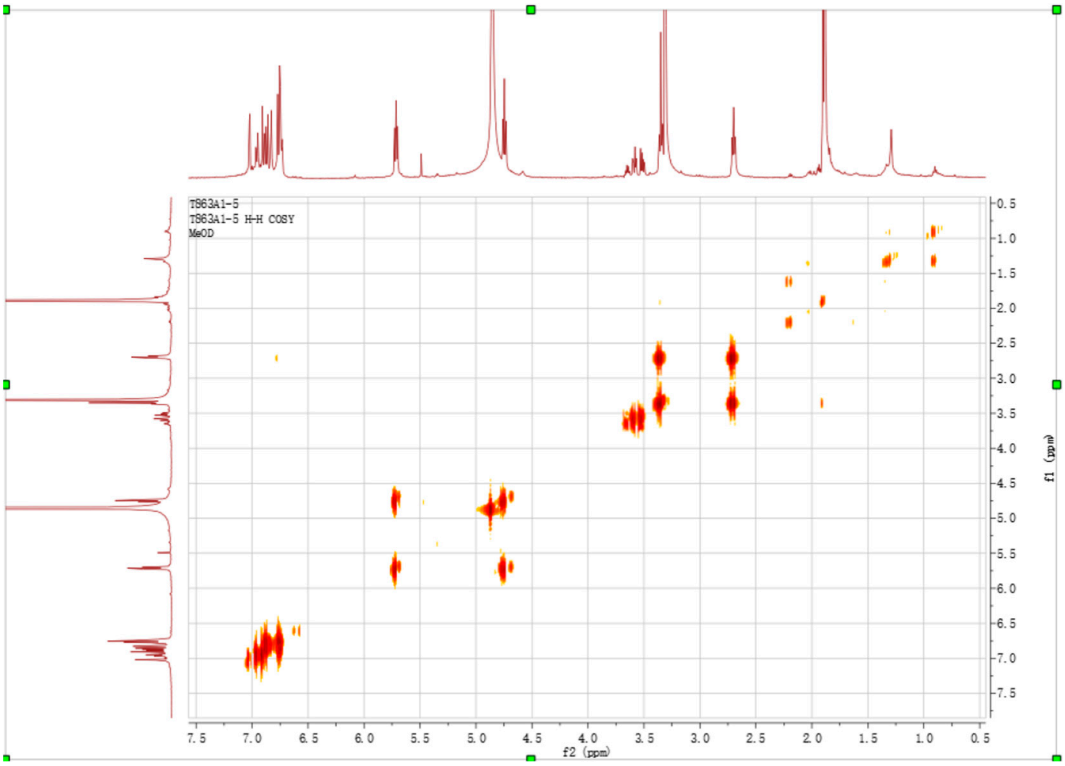

Figure S4. ^{1}H-^{1}H-COSY spectrum of **1** in methanol-d_4.

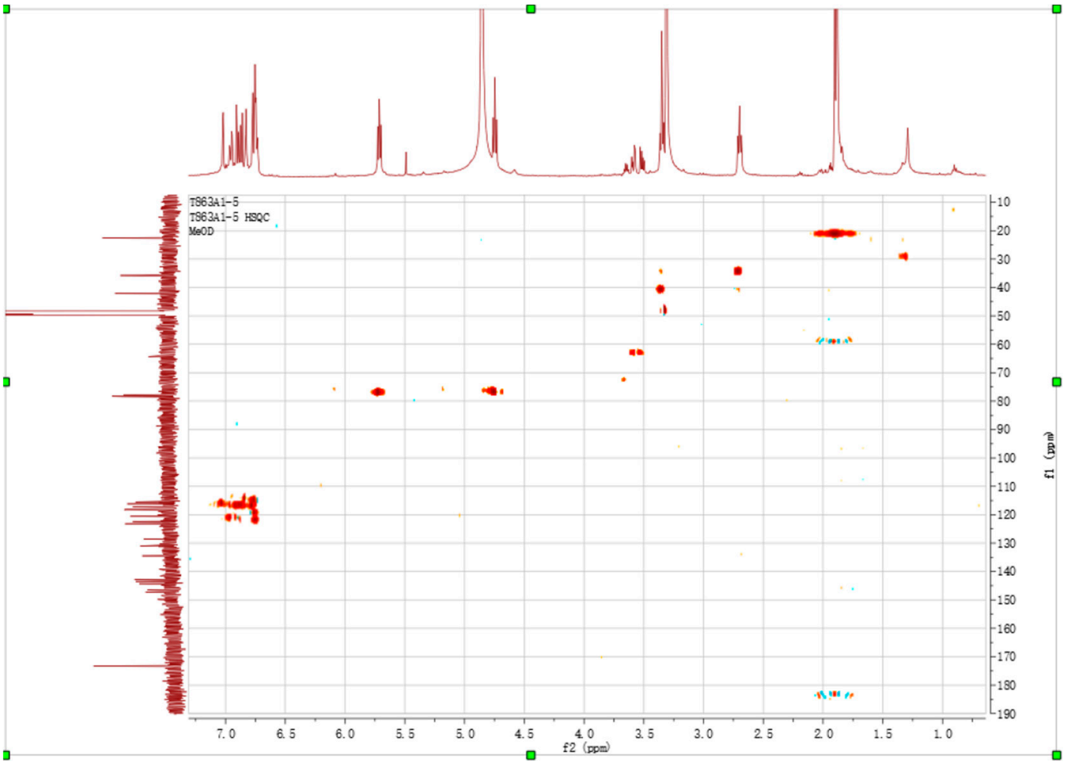

Figure S5. HSQC spectrum of **1** in methanol-*d*4.

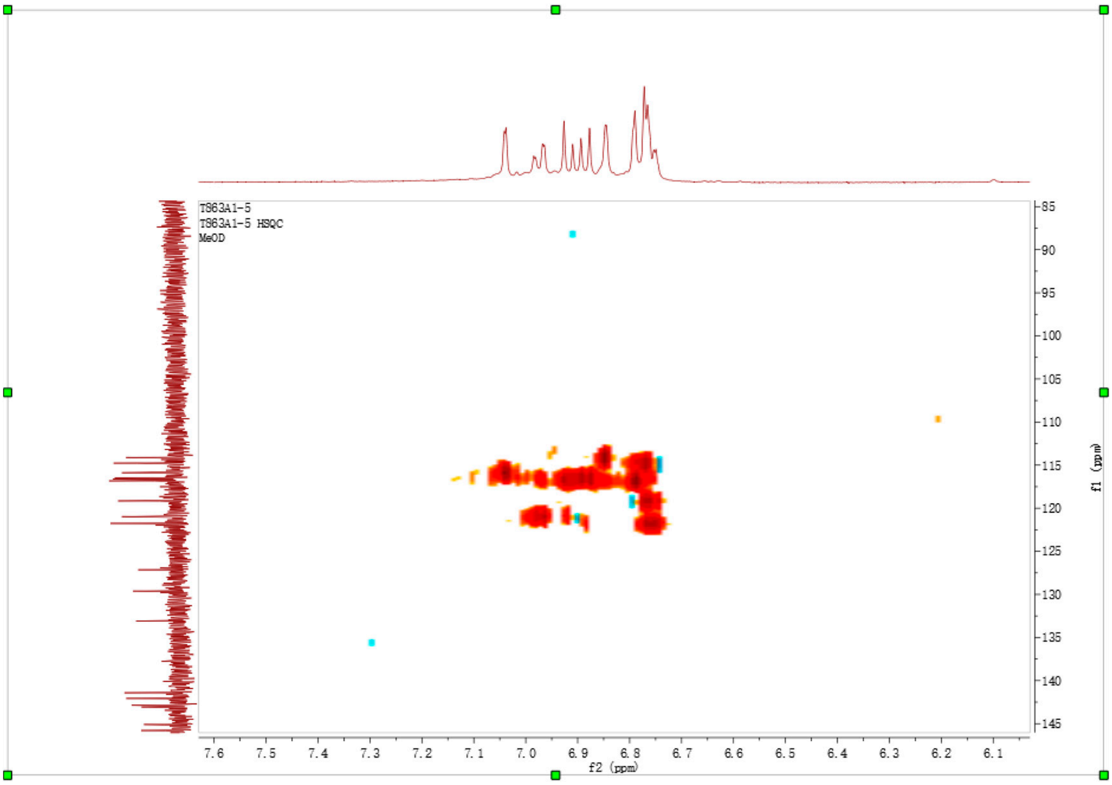

Figure S6. zoomed aromatic region of HSQC spectra of **1**.

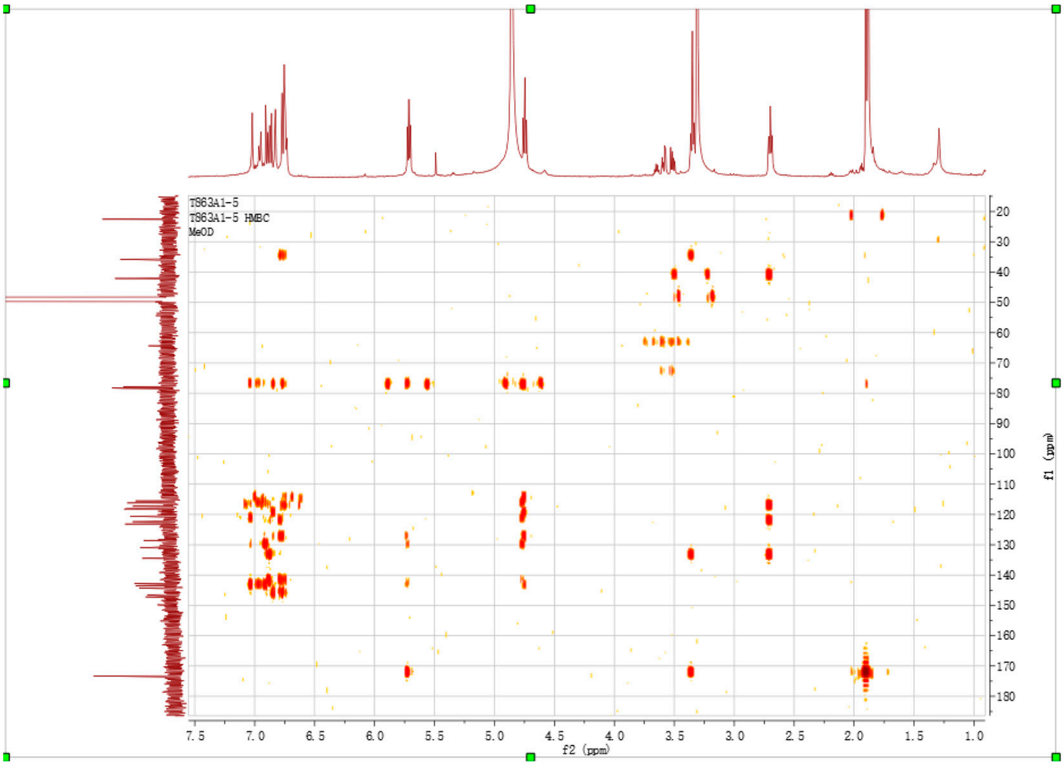

Figure S7. HMBC spectrum of **1** in methanol-*d*4.

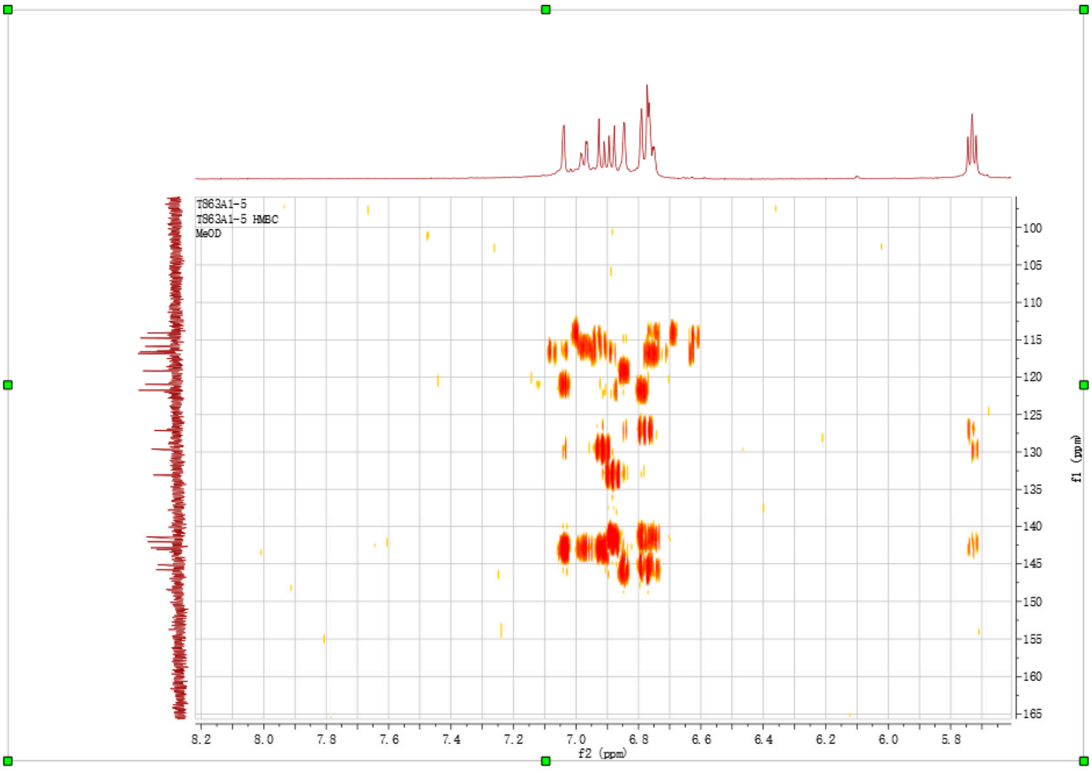

Figure S8. zoomed aromatic region of HMBC spectra of **1**.

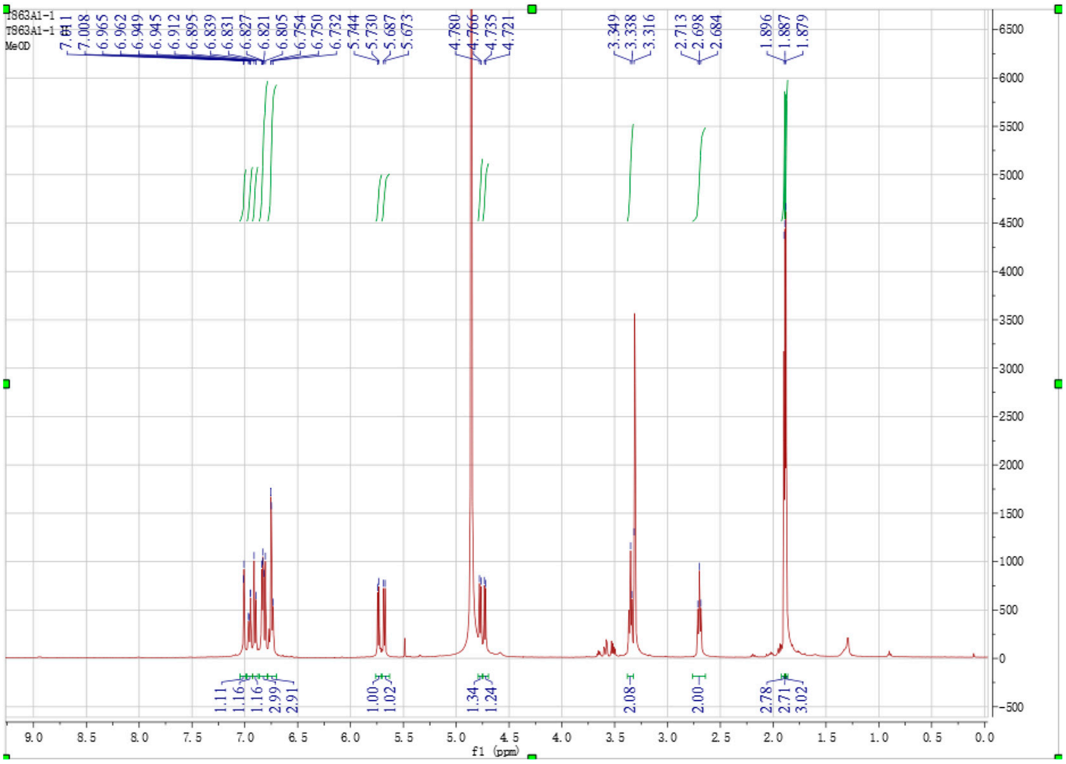

Figure S9. ^{1}H-NMR spectrum of **2** in methanol-d_4.

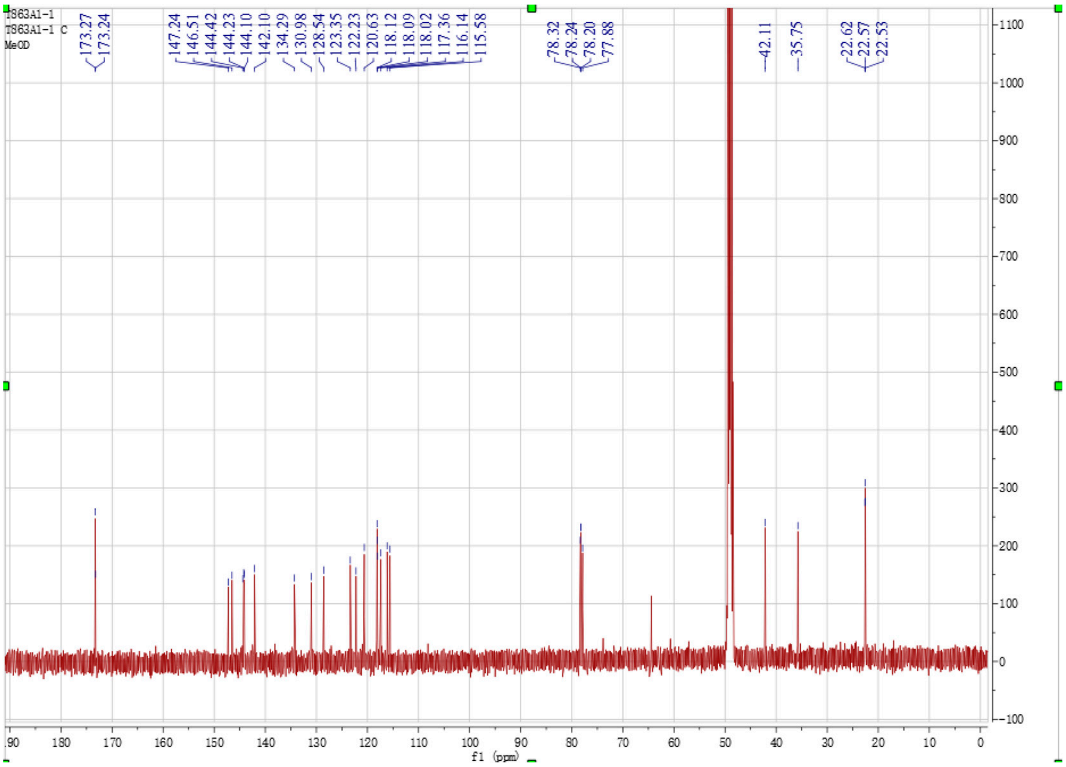

Figure S10. ^{13}C-NMR spectrum of **2** in methanol-d_4.

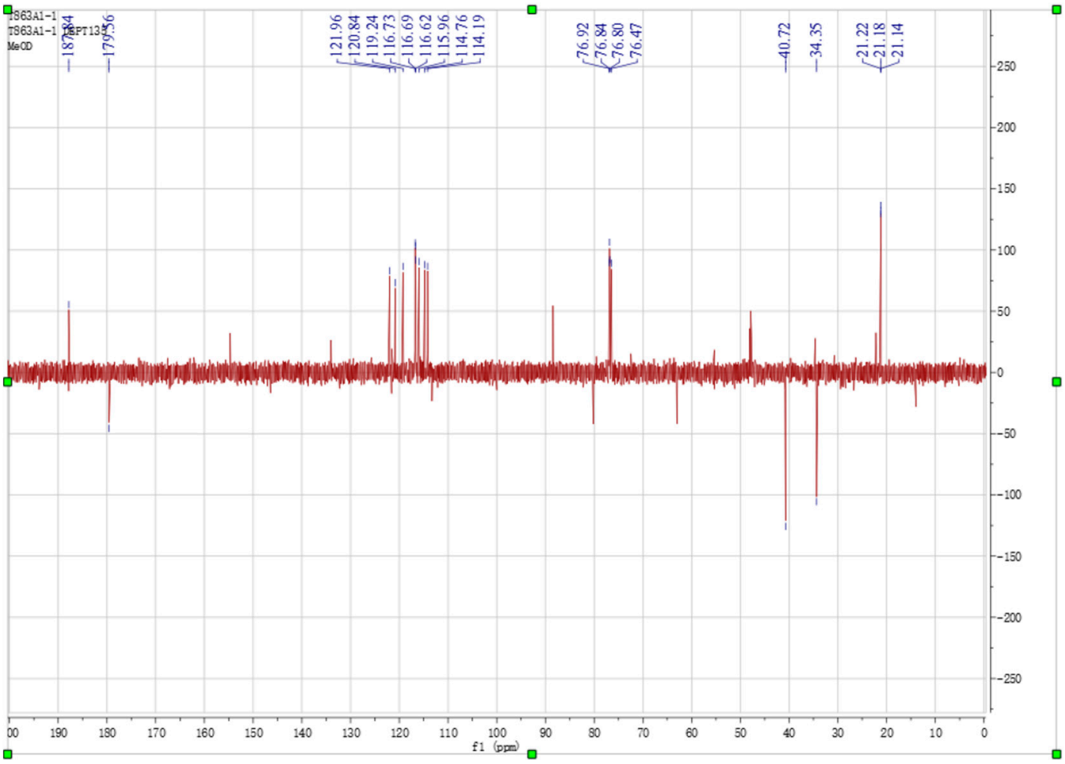

Figure S11. DEPT spectrum of **2** in methanol-d_4.

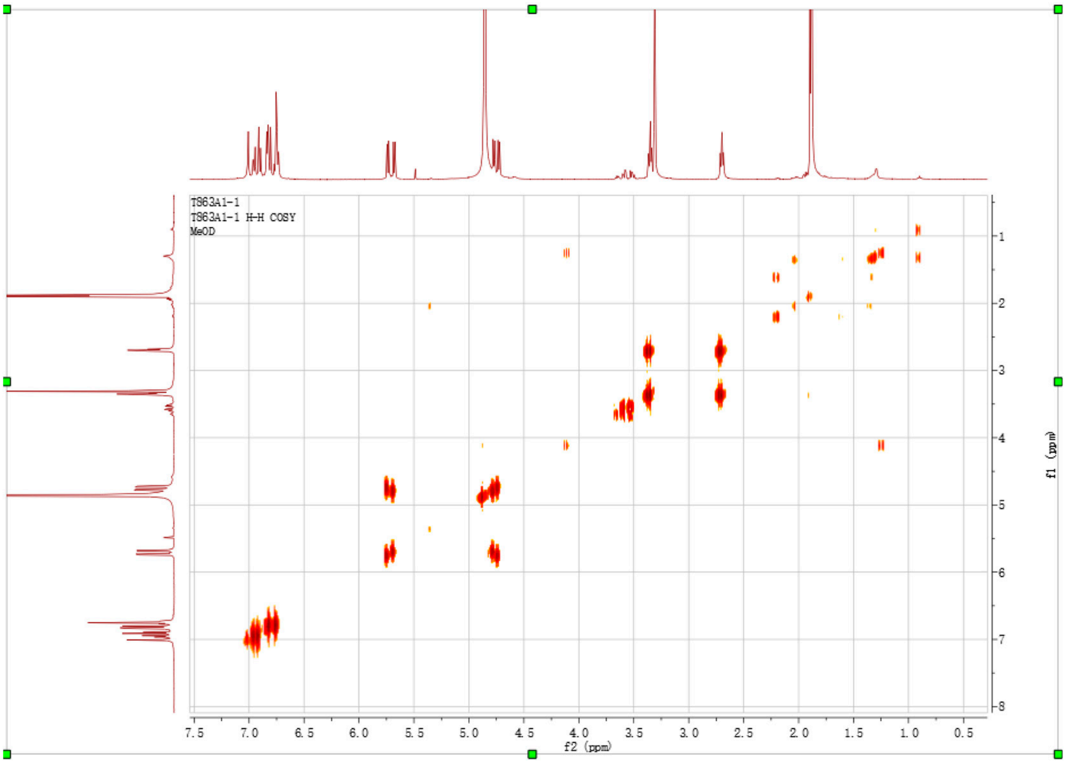

Figure S12. ^{1}H-^{1}H-COSY spectrum of **2** in methanol-d_4.

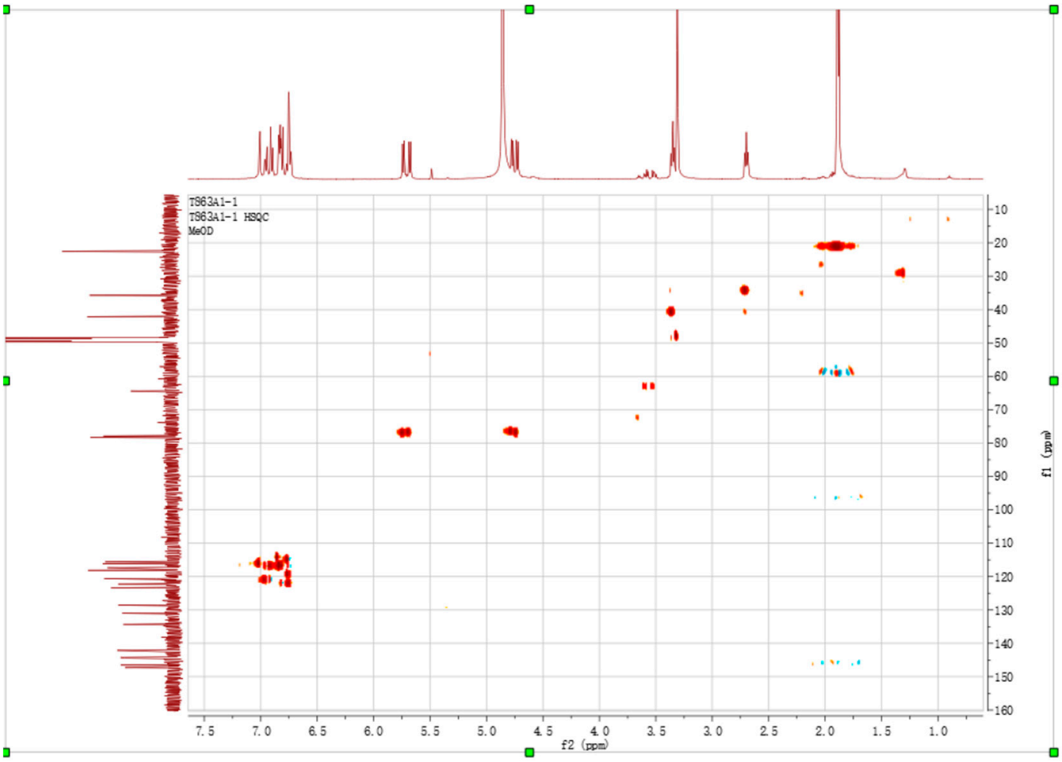

Figure S13. HSQC spectrum of **2** in methanol-d_4.

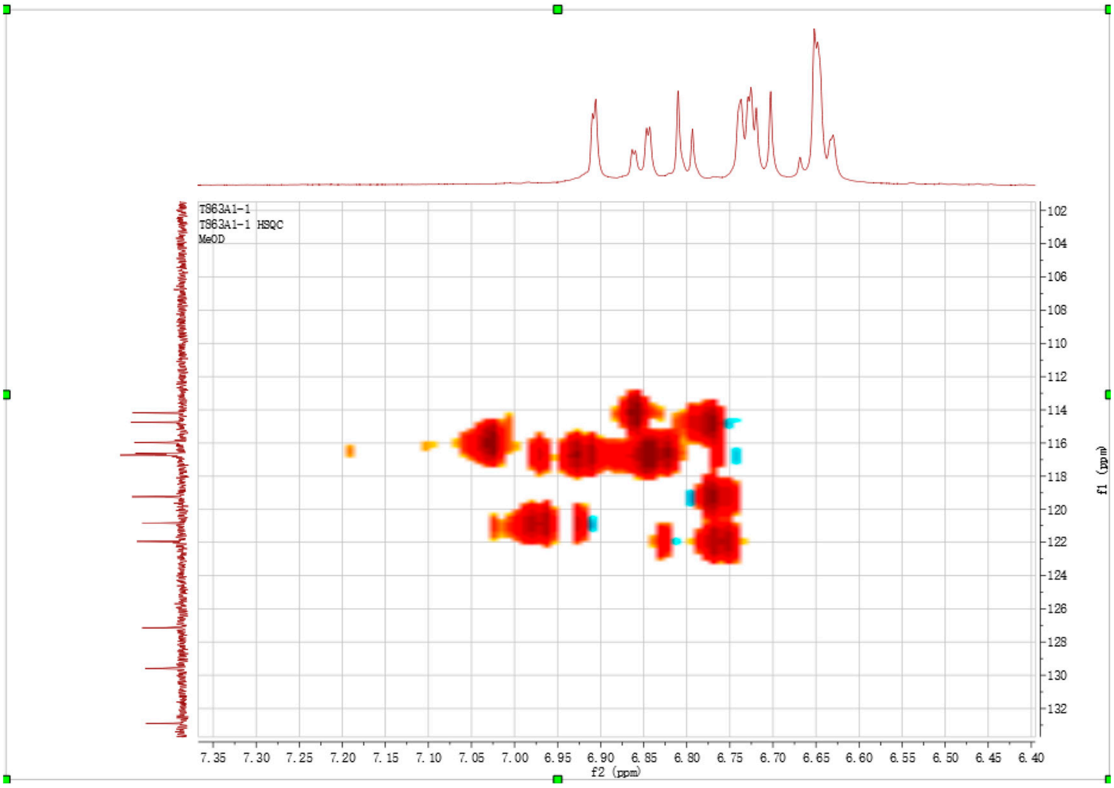

Figure S14. zoomed aromatic region of HSQC spectra of **2**.

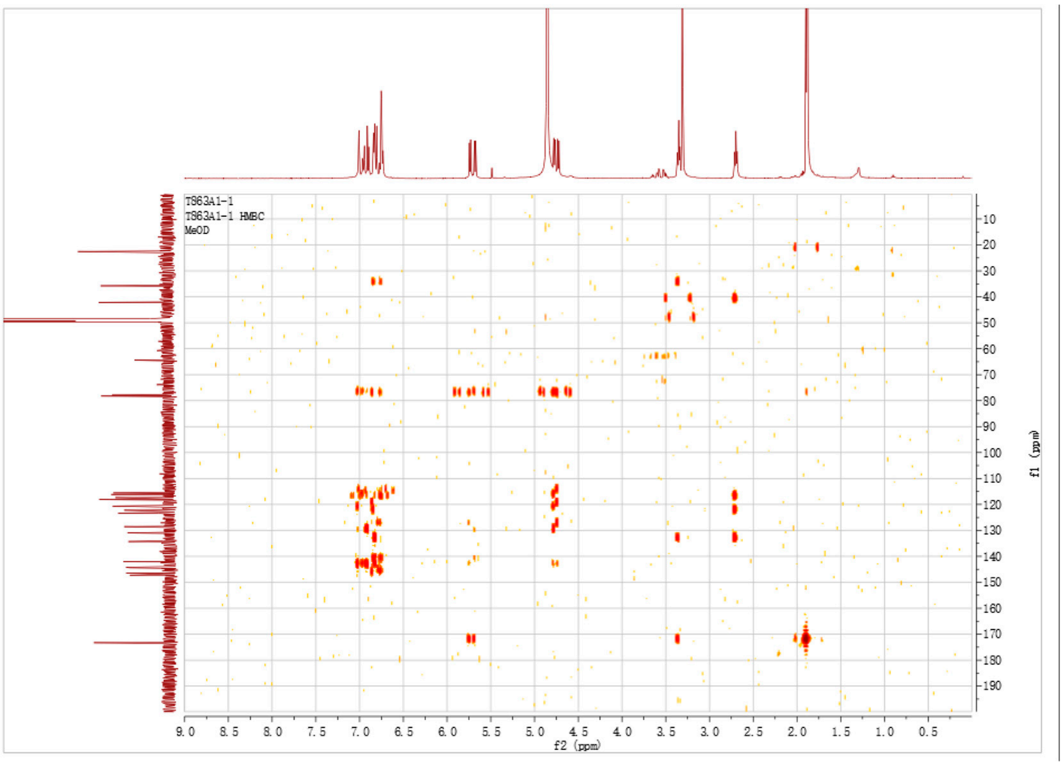

Figure S15. HMBC spectrum of **2** in methanol-d_4.

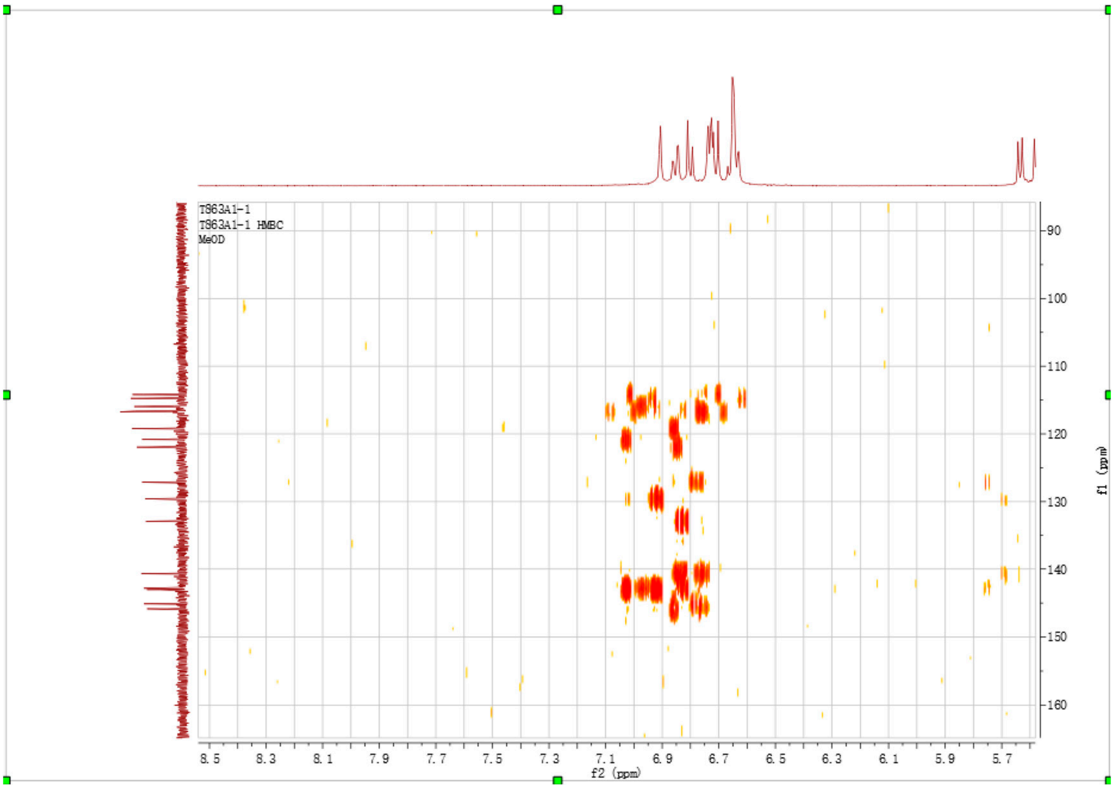

Figure S16. zoomed aromatic region of HMBC spectra of **2**.

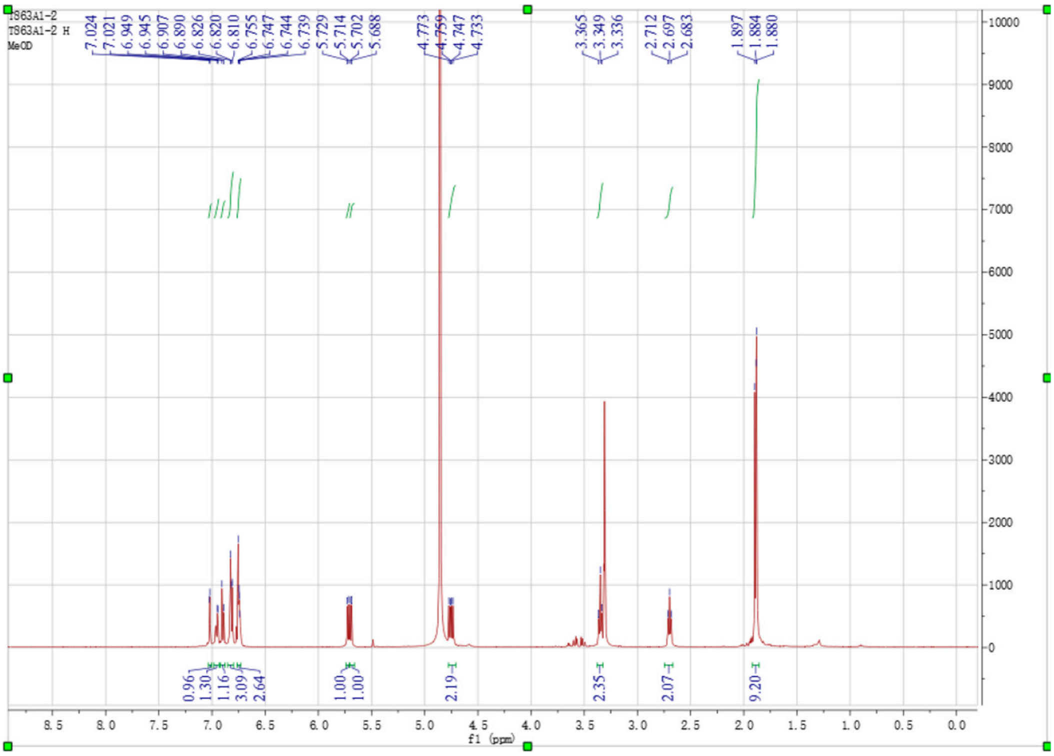

Figure S17. ¹H-NMR spectrum of **3** in methanol-*d*4.

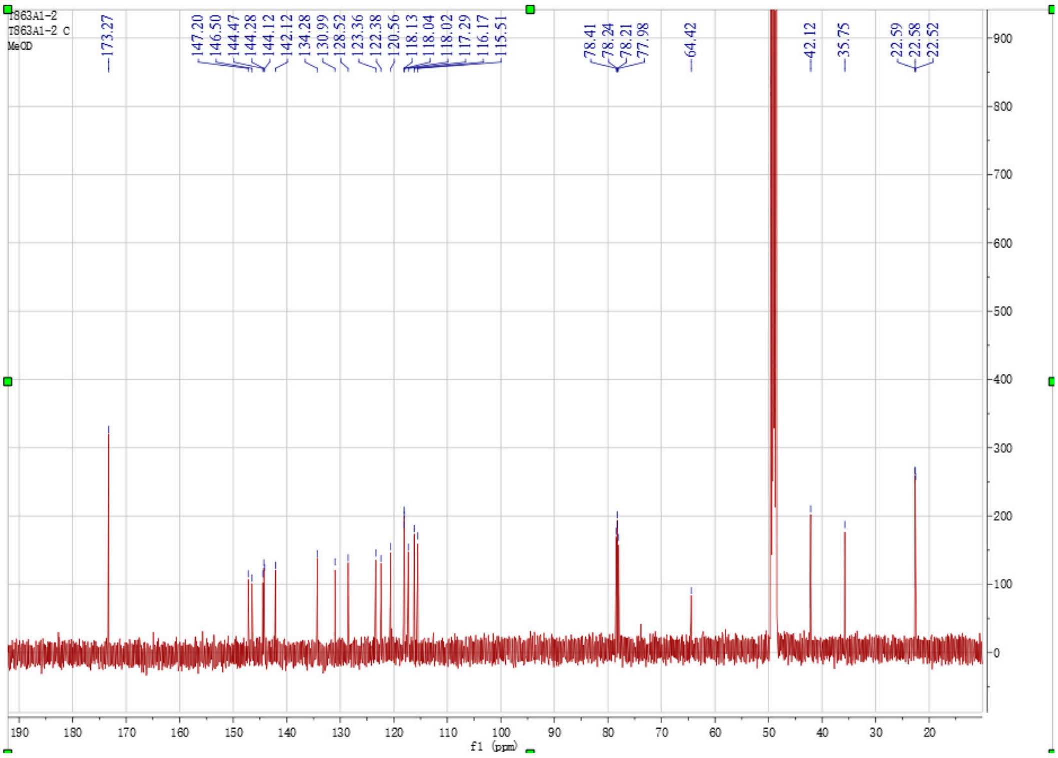

Figure S18. ¹³C-NMR spectrum of **3** in methanol-*d*4.

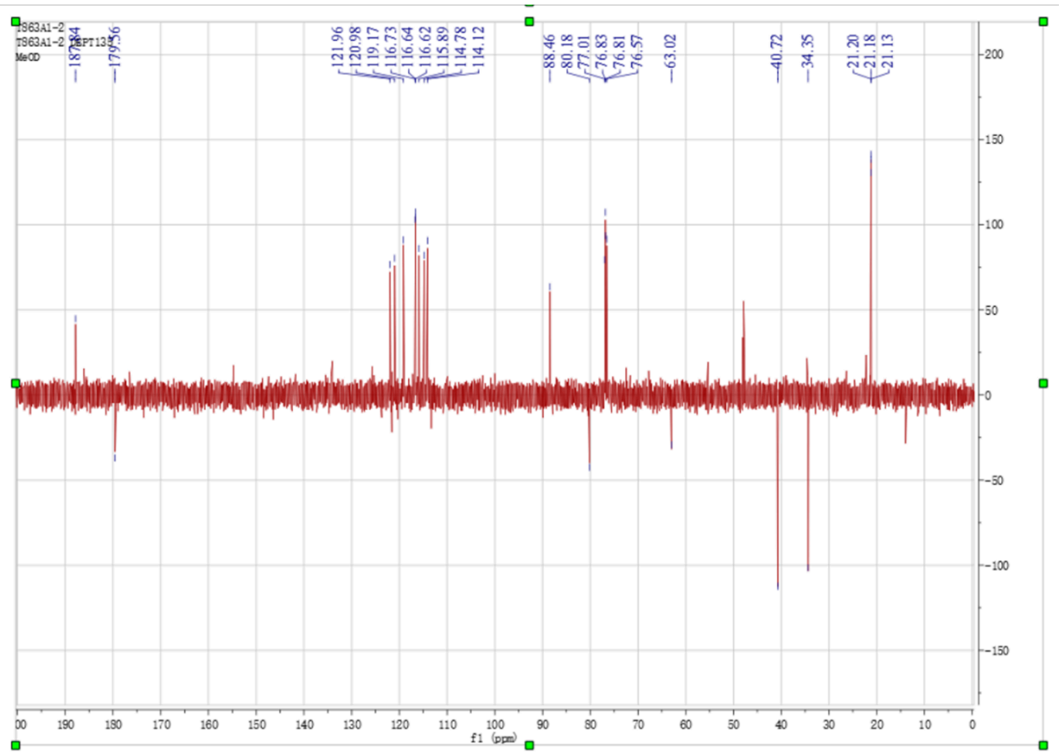

Figure S19. DEPT spectrum of **3** in methanol-d_4.

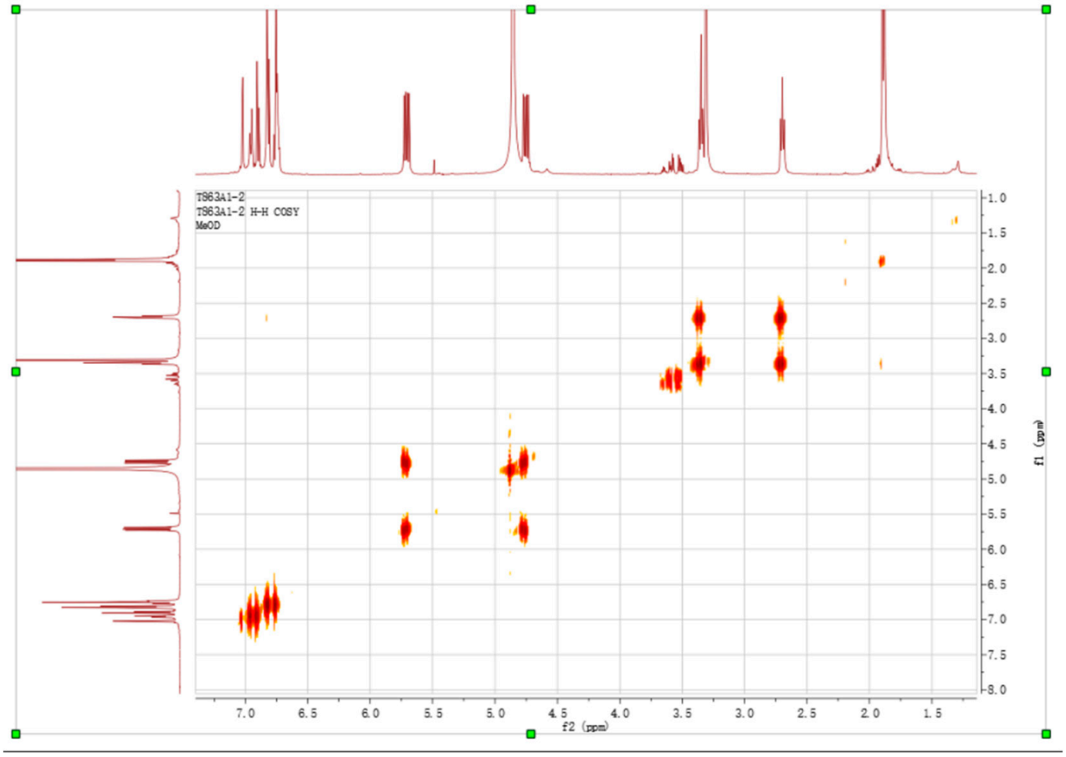

Figure S20. ^{1}H-^{1}H COSY spectrum of **3** in methanol-d_4.

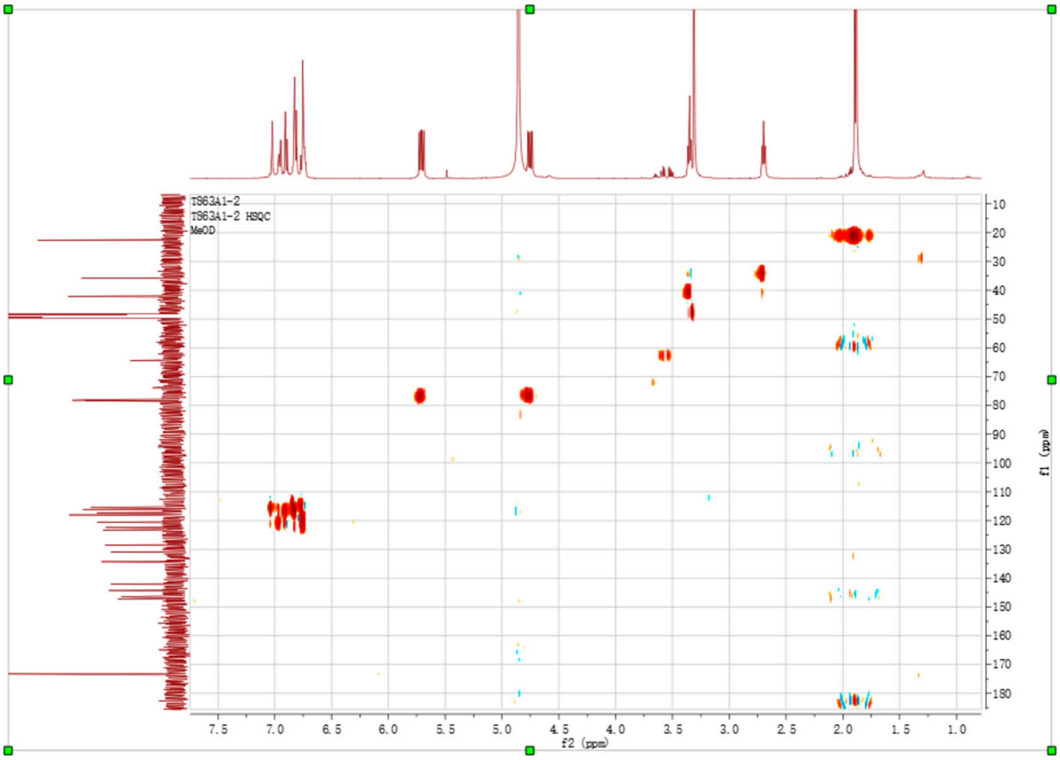

Figure S21. HSQC spectrum of **3** in methanol-d_4.

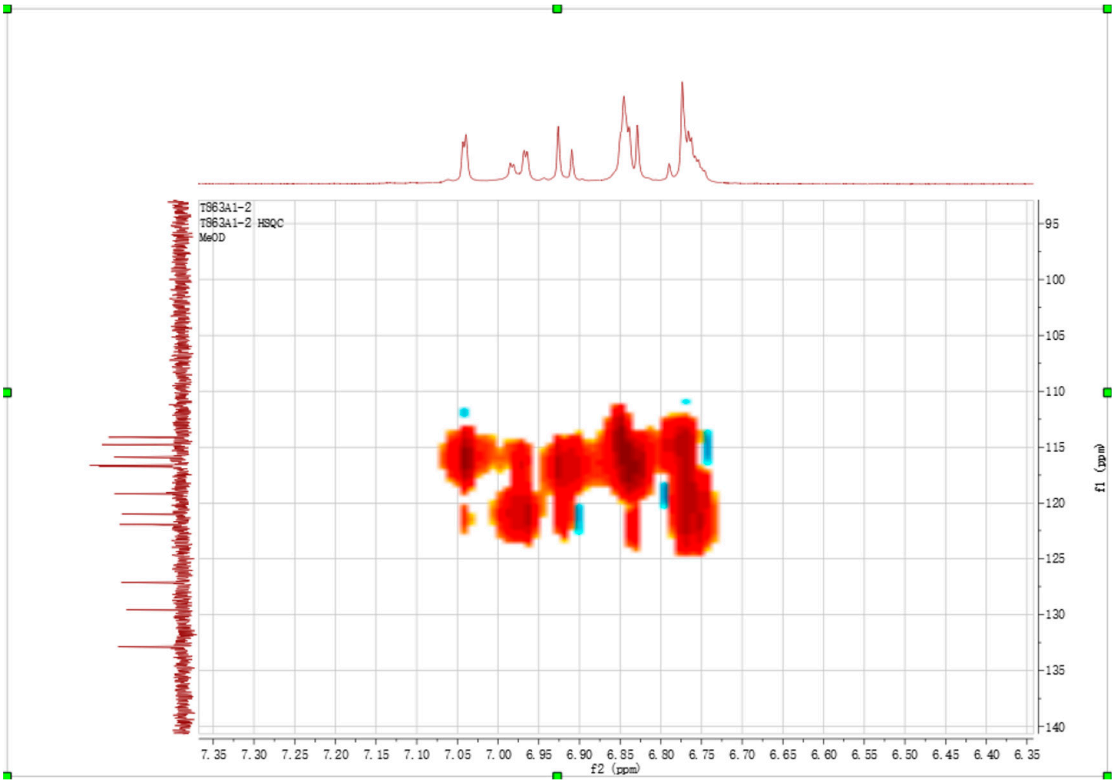

Figure S22. zoomed aromatic region of HSQC spectra of **3**.

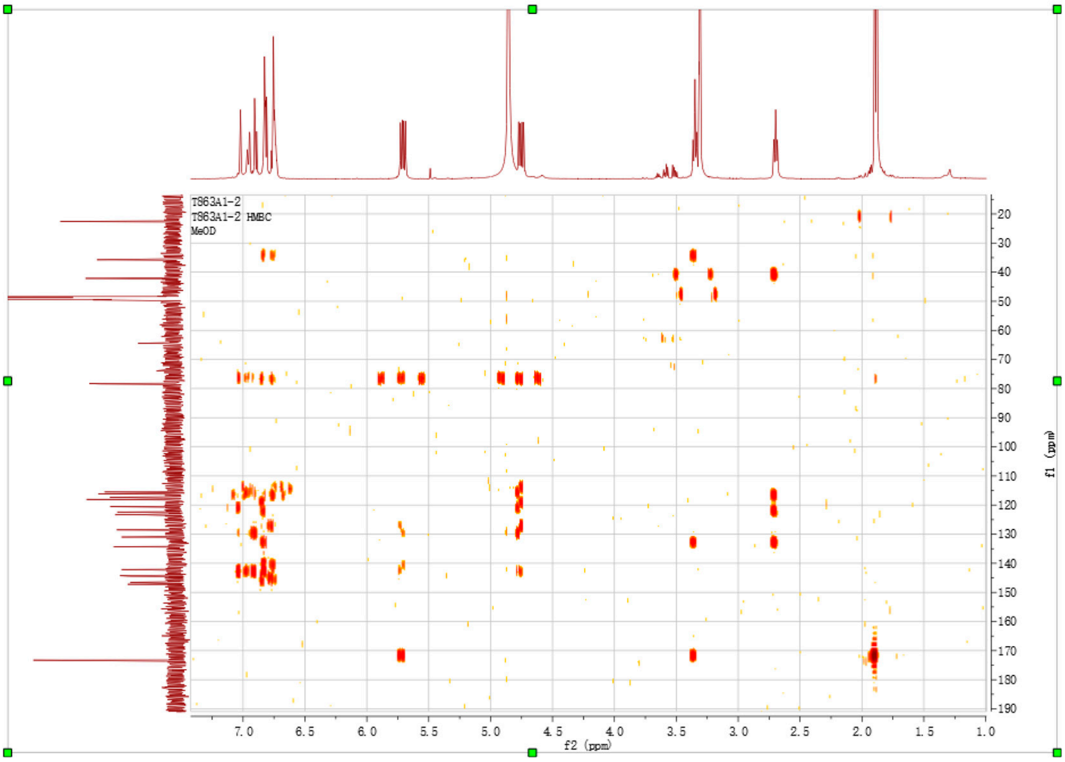

Figure S23. HMBC spectrum of **3** in methanol-d_4.

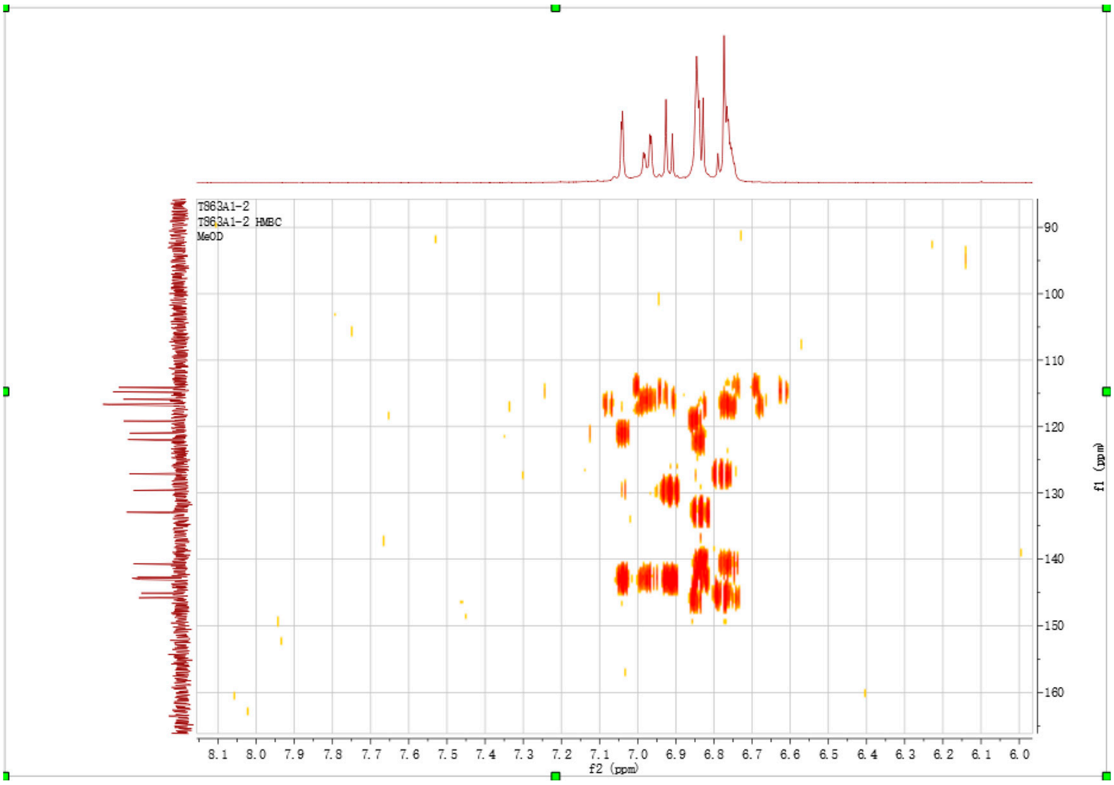

Figure S24. zoomed aromatic region of HMBC spectra of **3**.

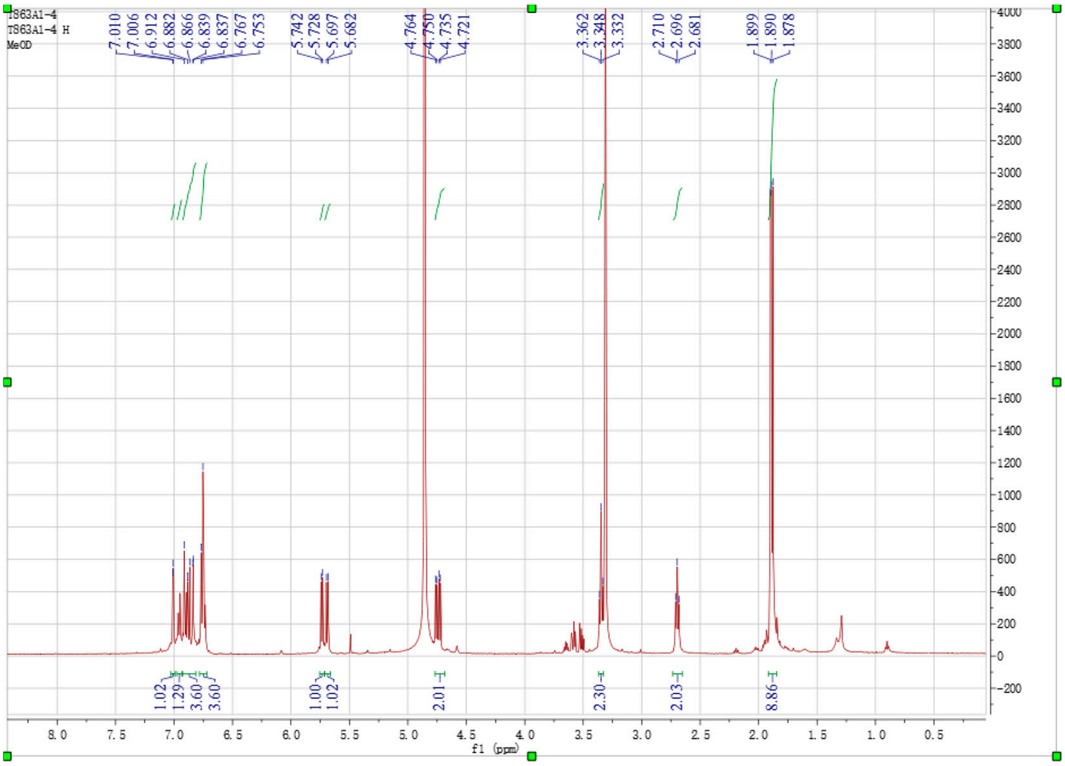

Figure S25. ^{1}H-NMR spectrum of **4** in methanol-d_4.

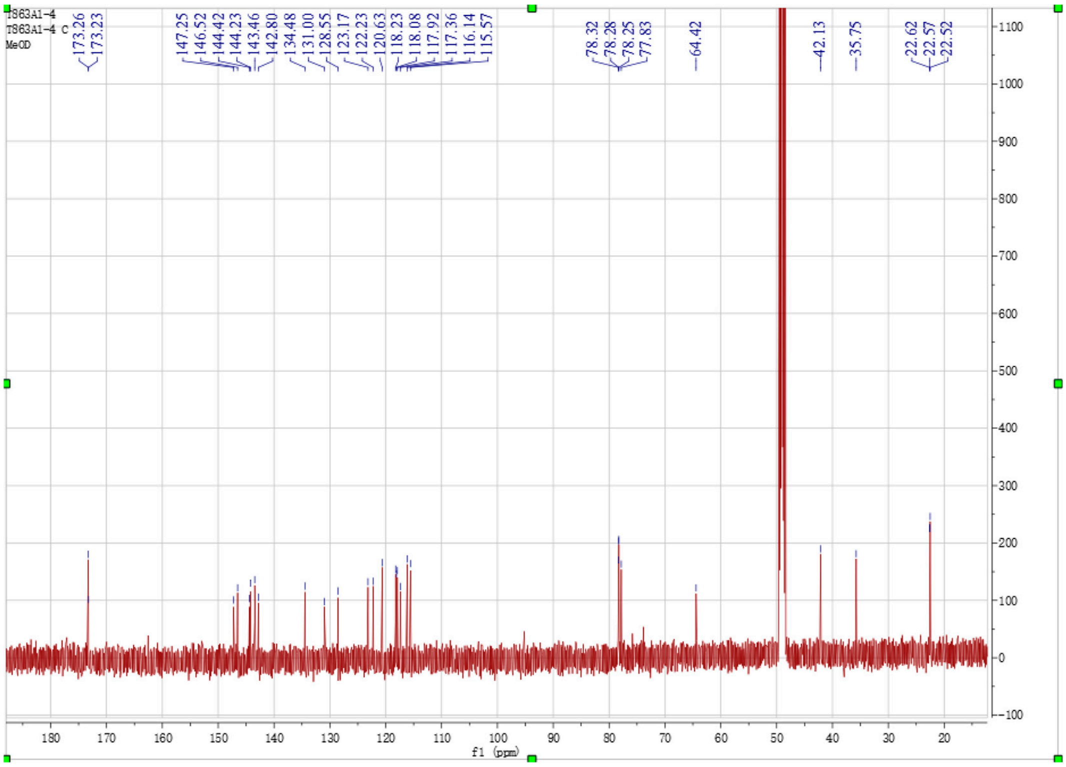

Figure S26. ^{13}C-NMR spectrum of **4** in methanol-d_4.

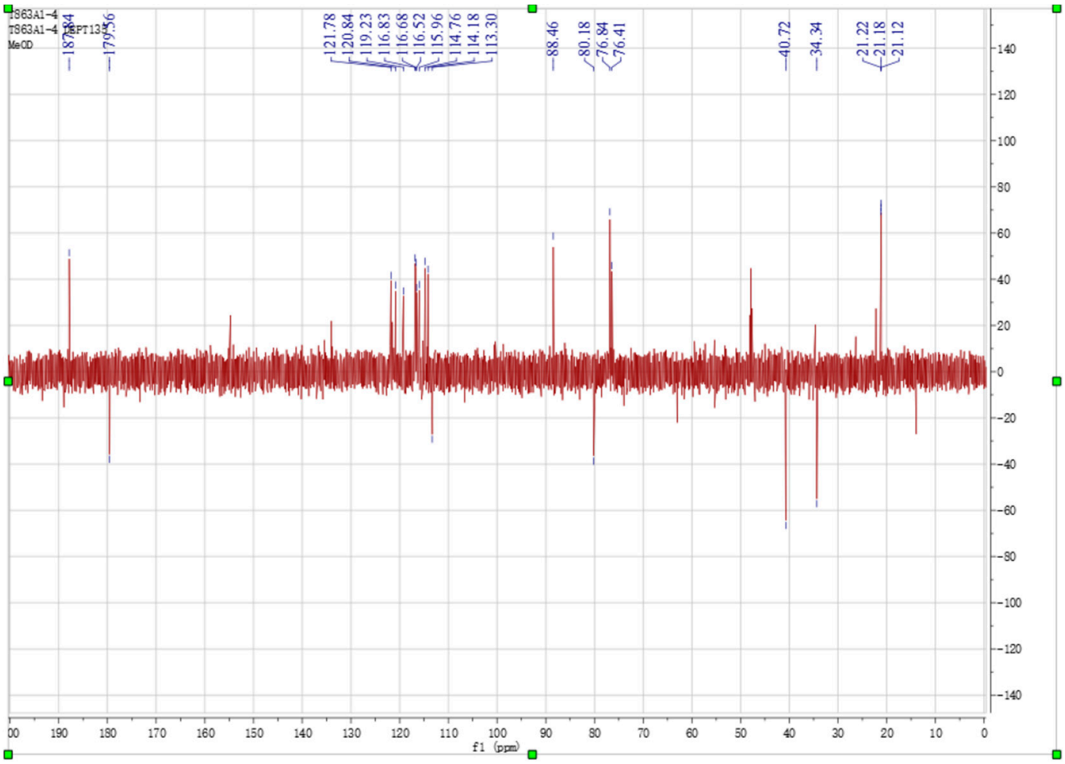

Figure S27. DEPT spectrum of **4** in methanol-d_4.

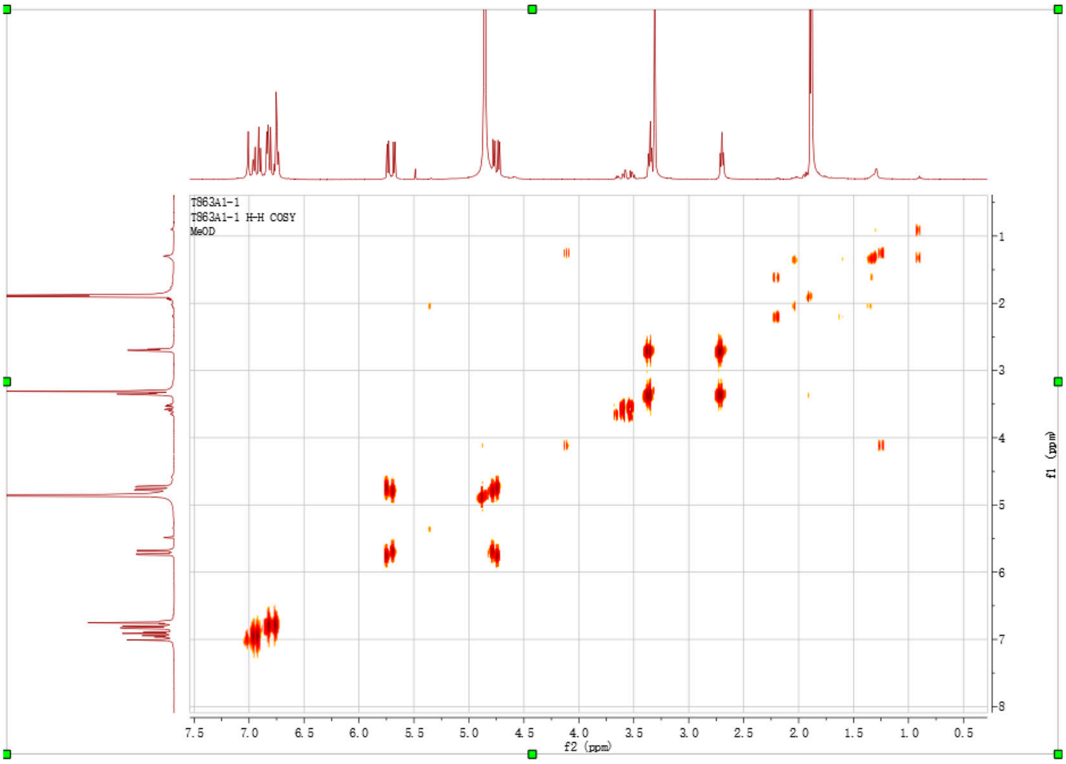

Figure S28. ^{1}H-^{1}H-COSY spectrum of **4** in methanol-d_4.

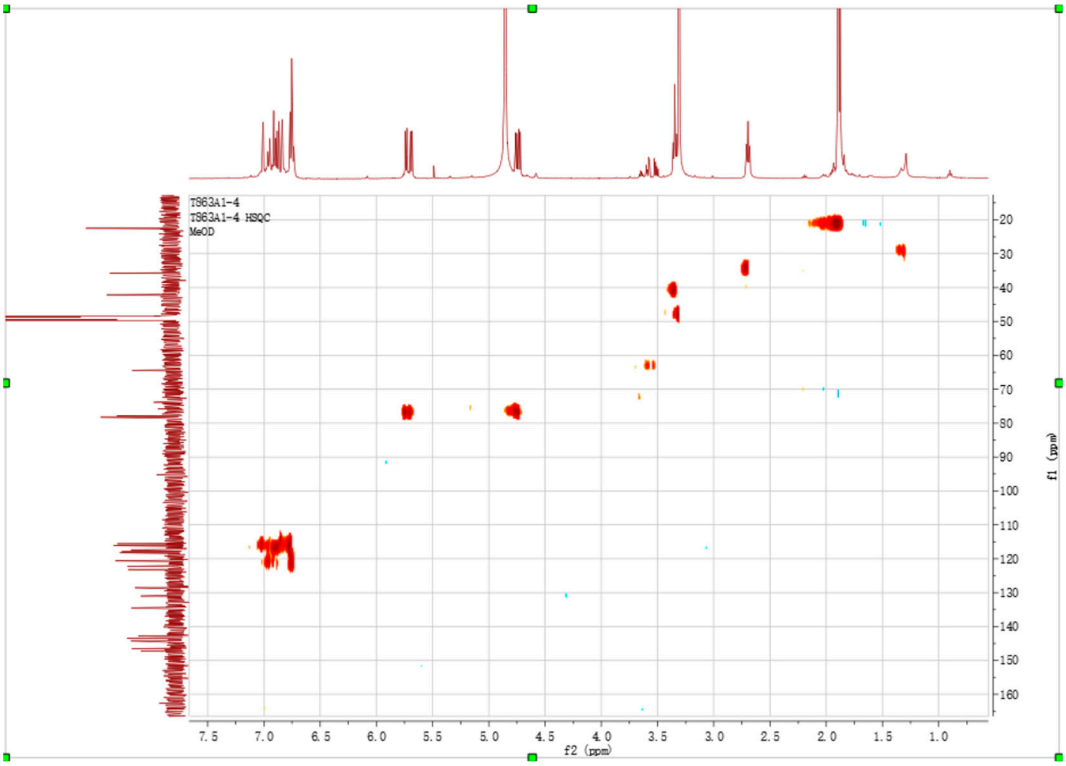

Figure S29. HSQC spectrum of **4** in methanol-d_4.

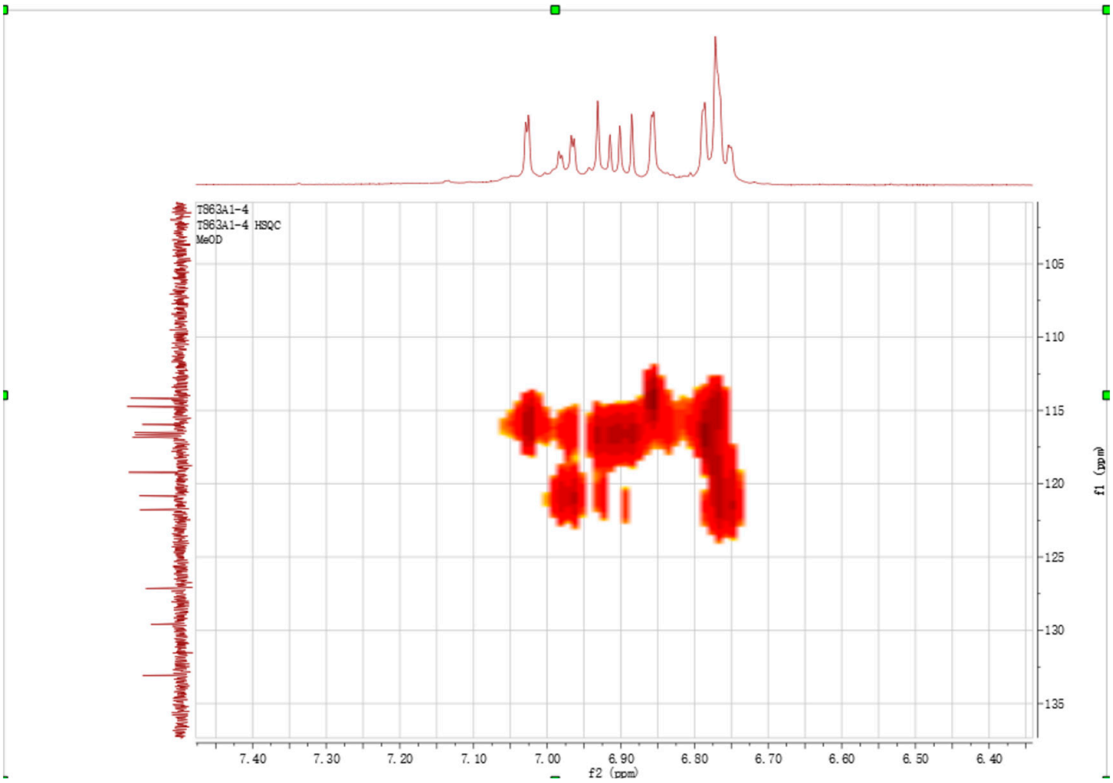

Figure S30. zoomed aromatic region of HSQC spectra of **4**.

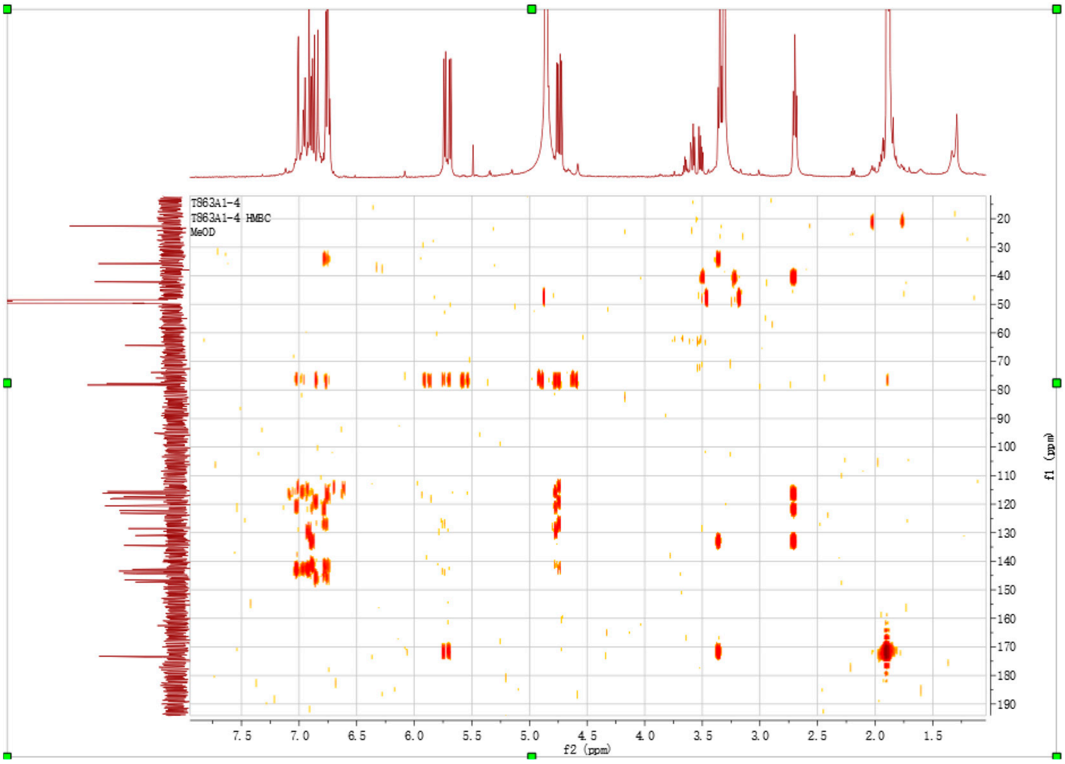

Figure S31. HMBC spectrum of **4** in methanol-d_4.

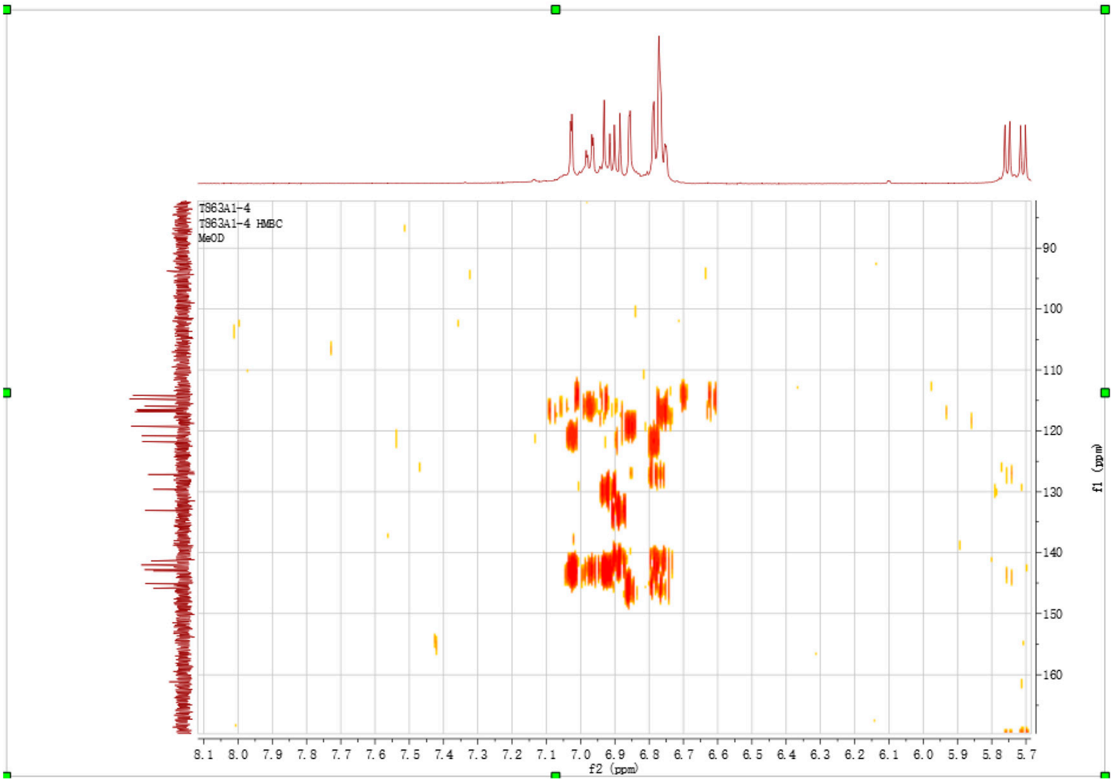

Figure S32. zoomed aromatic region of HMBC spectra of **4**.

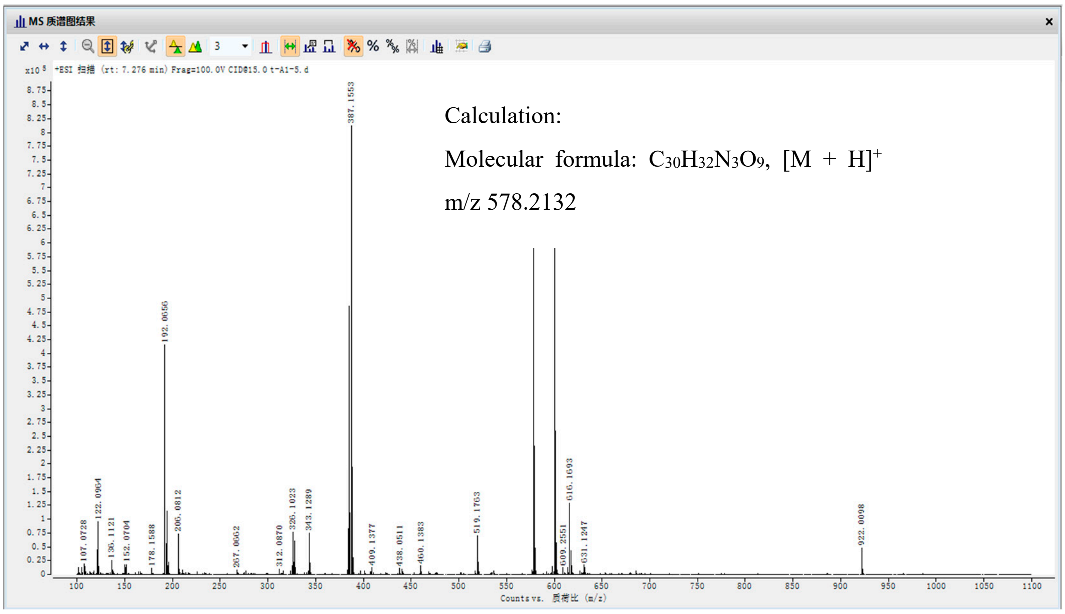

Figure S33. HR-ESI-MS of **1**.

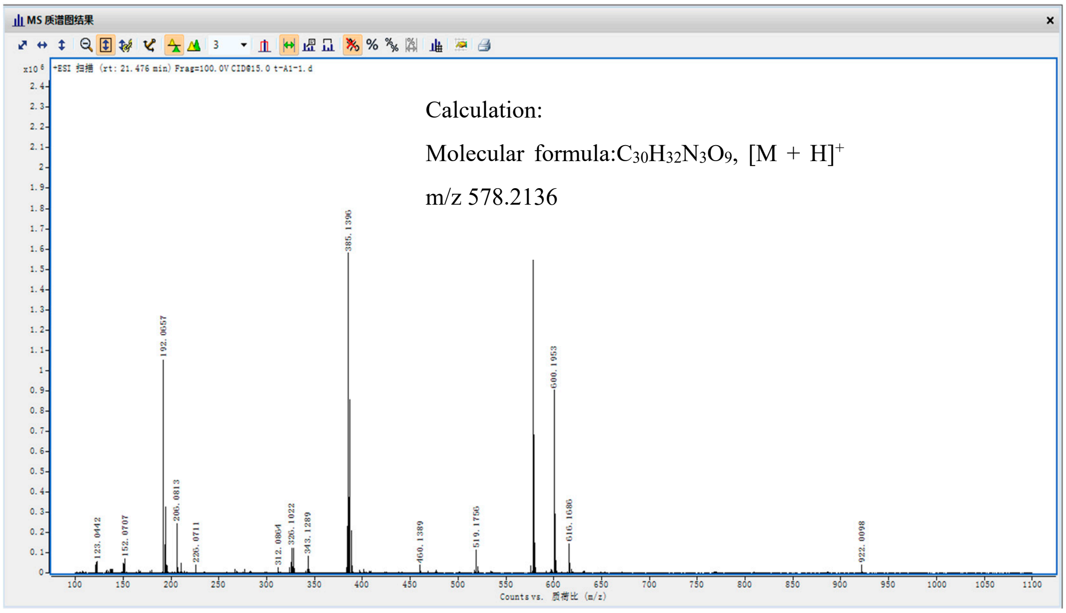

Figure S34. HR-ESI-MS of **2**.

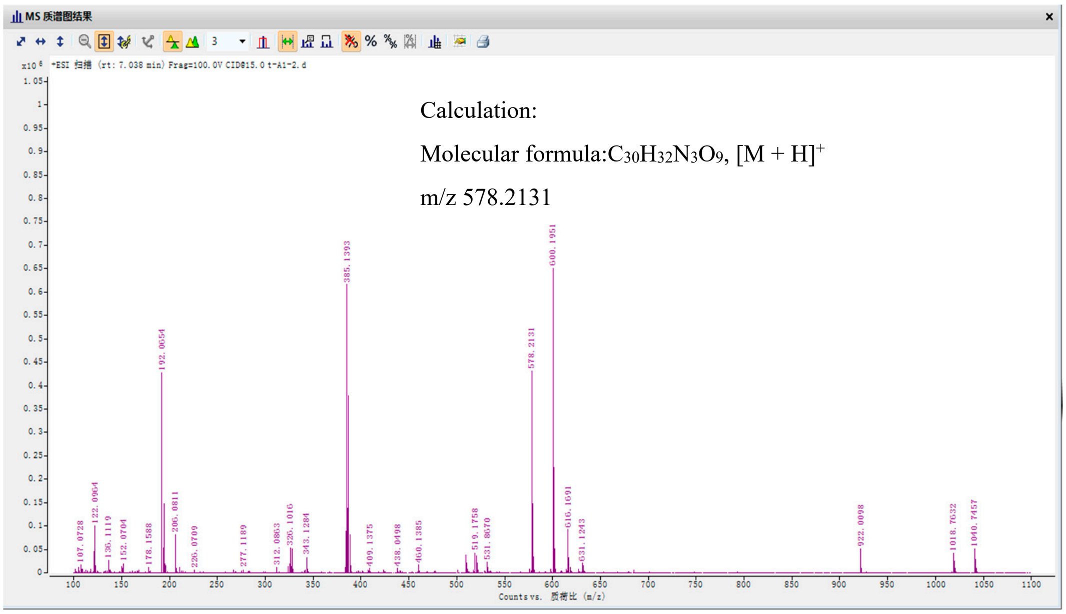

Figure S35. HR-ESI-MS of **3**.

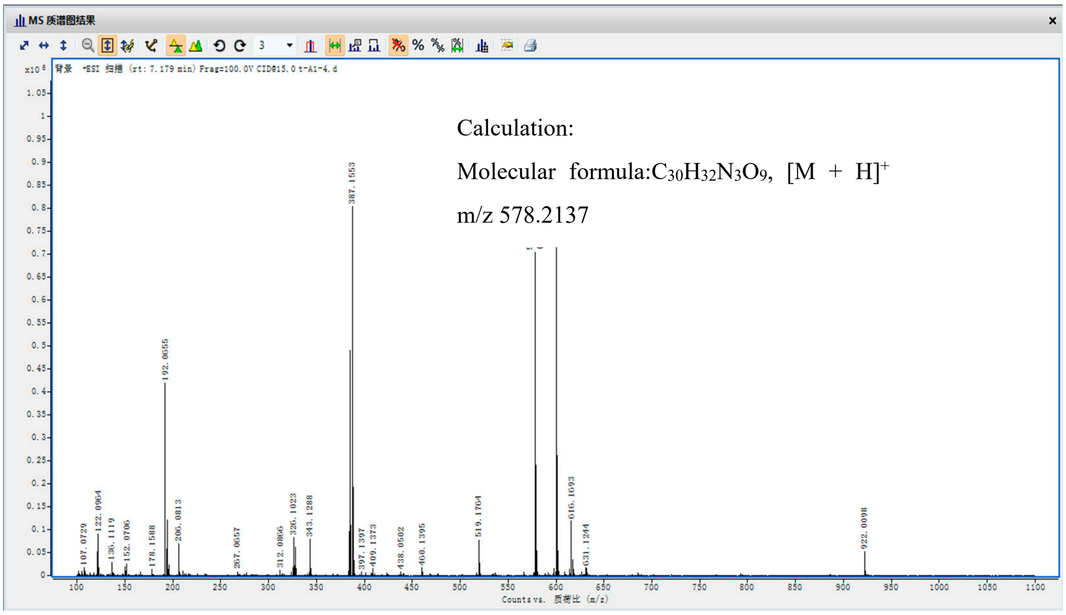

Figure S36. HR-ESI-MS of **4**.
